# Effect of transcutaneous auricular vagus nerve stimulation on impaired glucose tolerance: a pilot randomized study

**DOI:** 10.1186/1472-6882-14-203

**Published:** 2014-06-26

**Authors:** Feng Huang, Jianxun Dong, Jian Kong, Hongcai Wang, Hong Meng, Rosa B Spaeth, Stephanie Camhi, Xing Liao, Xia Li, Xu Zhai, Shaoyuan Li, Bing Zhu, Peijing Rong

**Affiliations:** 1 Institute of Acu-Mox, China Academy of Chinese Medical Sciences, 16# Nanxiao Street, Dongzhimennei, Beijing 100700, China; 2 Beijing University of Chinese Medicine, Beijing 100029, China; 3 Beijing Hospital of T.C.M Affiliated to Capital University of Medicine Sciences, Beijing 100010, China; 4 Department of Psychiatry, Massachusetts General Hospital, Harvard Medical School, Boston, MA, USA; 5 Department of Psychology, Endicott College, Beverly, MA, USA; 6 Institute of Basic Research in Clinical Medicine, China Academy of Chinese Medical Sciences, Beijing 100700, China

## Abstract

**Background:**

Impaired glucose tolerance (IGT) is a pre-diabetic state of hyperglycemia that is associated with insulin resistance, increased risk of type II diabetes, and cardiovascular pathology. Recently, investigators hypothesized that decreased vagus nerve activity may be the underlying mechanism of metabolic syndrome including obesity, elevated glucose levels, and high blood pressure.

**Methods:**

In this pilot randomized clinical trial, we compared the efficacy of transcutaneous auricular vagus nerve stimulation (taVNS) and sham taVNS on patients with IGT. 72 participants with IGT were single-blinded and were randomly allocated by computer-generated envelope to either taVNS or sham taVNS treatment groups. In addition, 30 IGT adults were recruited as a control population and not assigned treatment so as to monitor the natural fluctuation of glucose tolerance in IGT patients. All treatments were self-administered by the patients at home after training at the hospital. Patients were instructed to fill in a patient diary booklet each day to describe any side effects after each treatment. The treatment period was 12 weeks in duration. Baseline comparison between treatment and control group showed no difference in weight, BMI, or measures of systolic blood pressure, diastolic blood pressure, fasting plasma glucose (FPG), 2-hour plasma glucose (2hPG), or glycosylated hemoglobin (HbAlc).

**Results:**

100 participants completed the study and were included in data analysis. Two female patients (one in the taVNS group, one in the sham taVNS group) dropped out of the study due to stimulation-evoked dizziness. The symptoms were relieved after stopping treatment. Compared with sham taVNS, taVNS significantly reduced the two-hour glucose tolerance (*F*(2) = 5.79, p = 0.004). In addition, we found that taVNS significantly decreased (*F*(1) = 4.21, p = 0.044) systolic blood pressure over time compared with sham taVNS. Compared with the no-treatment control group, patients receiving taVNS significantly differed in measures of FPG (*F*(2) = 10.62, p < 0.001), 2hPG *F*(2) = 25.18, p < 0.001) and HbAlc (*F*(1) = 12.79, p = 0.001) over the course of the 12 week treatment period.

**Conclusions:**

Our study suggests that taVNS is a promising, simple, and cost-effective treatment for IGT/ pre-diabetes with only slight risk of mild side-effects.

## Background

Pre-diabetes, defined as impaired glucose tolerance (IGT), or impaired fasting glucose (IFG) or both, represents an intermediate state between normal glucose homeostasis and diabetes [1]. IGT is a pre-diabetic state of hyperglycemia that is associated with insulin resistance and increased risk of cardiovascular pathology. In patients with IGT, the main site of insulin resistance is muscle tissue; this is in contrast to another state of hyperglycemia, IFG, in which the site of insulin resistance is the liver [1]. IGT affects about 11% of people aged 20–74 years in the United States [2]and 17% of those aged 40–65 years in Britain [3]. The American Diabetes Prevention Program (DPP) showed that approximately 10% of individuals with IGT developed diabetes on an annual basis [4]. In another study, investigators found that 30% of patients with IGT developed diabetes over a period of 8 years [5]. In a 10 year follow up study, results showed that 15% of people with IGT developed non-insulin dependent diabetes and 22% remained glucose intolerant [6]. Although patients with transient IGT can revert to normal, they remain at increased long term risk of developing non-insulin dependent diabetes. By the time they develop diabetes, 50% will already have established complications, 16% coronary artery disease, and 30% retinopathy [7].

Current opinion suggests that pre-diabetes should be treated either with lifestyle interventions, which target obesity and physical inactivity, and/or with pharmacological intervention based on anti-diabetic drugs, in order to prevent progression to diabetes [1]. For both interventions, compliance can be a serious problem, and the long term effects of pharmacological interventions remain to be tested [8].

In recent years, investigators have proposed the hypothesis that adequate vagus nerve activity can reduce the risk of metabolic syndromes such as obesity, elevated glucose level and blood pressure [9]. Thus, increasing the activity and firing of the vagus nerve by direct stimulation may prevent metabolic syndromes such as obesity, and elevated glucose levels and blood pressure [9]. However, the involvement of surgery, the perioperative risks, and the potentially significant side effects associated with surgery have limited the application of classic vagus nerve stimulation methods in the patient population.

More recently, investigators have implemented transcutaneous auricular vagus nerve stimulation (taVNS) in replacement of the classic VNS method to treat disorders such as epilepsy [10] and depression [11]. The rationale for using taVNS is that anatomical studies have shown that the ear is the only place on the surface of the human body where there is afferent vagus nerve distribution [12]. Thus, direct stimulation of the afferent vagus nerve fibers on the ear may produce an effect similar to classic VNS without the burden of surgical intervention [13].

In this study, we performed a pilot randomized clinical trial to test the efficacy of taVNS in IGT patients.

## Methods

This study was registered at the Chinese Cochrane Centre, International Clinical Epidemiology Network Resource and Training Center (ChiCTR-TRC-12002522). The Institutional Ethics Committee of the China Academy of Chinese Medical Sciences approved this study. All clinical investigative procedures were conducted according to the principles expressed in the Declaration of Helsinki. All patients and healthy controls signed a consent form prior to initiation of study procedures.

### Study population

IGT outpatients were recruited from the China Academy of Chinese Medical Sciences acupuncture hospital for a randomized clinical trial to test the efficacy of taVNS. Subject recruitment and all study procedures were completed between November 2009 and December 2010.

In order to monitor the natural fluctuation of glucose tolerance in IGT patients, an additional 30 IGT patients were recruited from free clinics in the community through advertisements in the local newspaper and posters. Patients in the no-treatment control group were required to fulfill the same inclusion criteria as patients who were randomized to receive treatment. Recruitment for the control population took place during the same period of time as for the two treatment groups. The control population was included to explore the variation of patient glucose levels under normal life conditions for 12 weeks without treatment.

All patients met the World Health Organization (WHO) standard diagnosis of IGT [14], and additionally were required to meet all of the following inclusion criteria:

a. Patients between 20 and 70 years of age

b. Levels of FPG less than 7.0 mmol/L and levels of 2hPG between 7.8 and 11.1 mmol/L at the time of enrollment

c. Ability and willingness to provide informed consent

d. No history of serious infections in the previous month.

Individuals were excluded from participation if they fulfilled any of the following exclusion criteria:

a. Risk of serious cardiovascular and hematopoietic system disease, or other serious primary diseases or mental illnesses

b. Pregnant or lactating women

c. Use of hypoglycemic western medicine or insulin treatment

d. Failure to cooperate during the treatment period

e. History of alcohol dependency or use of illicit drugs.

### Randomization

This study was designed as a single blind randomized controlled trial (RCT). After screening, 72 IGT participants were randomly assigned to either the transcutaneous auricular vagus nerve stimulation group (taVNS) or sham taVNS group (randomization sequence created using SAS System 7.0). All randomization assignments were pre-packed in envelopes and consecutively numbered for each participant according to the randomization schedule. All participants were blinded to their treatment modality and received the randomly-assigned intervention, as described in the envelope, until the end of the study.

### Intervention

After group randomization, all patients received training at the hospital regarding how to use the Transcutaneous Electrical Nerve Stimulator device to apply stimulation. All subsequent treatments were self-administered by the patients at home. Patients were also instructed to complete a patient diary booklet each day to describe any side effects corresponding with or temporally related to treatment. The investigators checked all booklets at the 6 and 12-week assessments.

### taVNS treatment

#### Location

**Figure 1:**
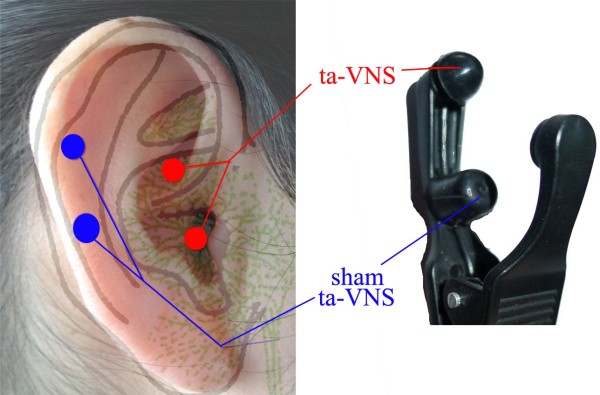
**Location of taVNS and sham taVNS. **Red dots indicate locations of taVNS, blue dots indicate the location of sham taVNS.

The points for taVNS are located in the auricular concha area where there is rich vagus nerve branch distribution (Figure 1).

### Intervention procedure

**Figure 2:**
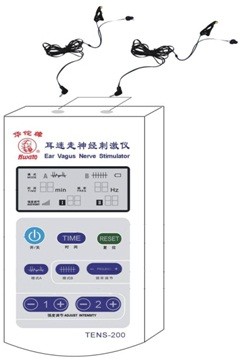
Ear vagus nerve stimulator used for taVNS.

taVNS was applied using a Huatuo ear vagus nerve stimulator (TENS-200) developed by Suzhou manufacture of Medical Device and Material (see Figure 2). Stimulation parameters for both the taVNS and sham taVNS groups were 1 mA of electrical current at a frequency of 20 Hz with pulse duration ≤ 1 ms, administered twice daily. The intensity was adjusted based on the individual tolerance of patients. Each postprandial treatment lasted 20 minutes and was applied half of an hour after eating. After the training session, all subsequent treatments were applied independently by the patient at home.

### Sham taVNS treatment

#### Location

The stimulation points for sham taVNS are located at the superior scapha (the midpoint of the outer ear margin), where we expect no vagus nerve distribution (Figure 1) based on previous studies [12].

### Intervention procedure

All procedures performed in the sham taVNS treatment group were identical to those performed for the taVNS group, with the exception of the altered location of stimulations.

### No-treatment control

All patients in the no-treatment control group were recruited from community free clinics that provide free physical exams, including blood glucose testing. No taVNS or sham taVNS treatments were applied.

### Outcome measurements

The primary outcome measure of this study is the change in 2hPG levels measured at baseline, 6-weeks, and 12-weeks. Secondary outcomes include changes in FPG levels, HbAlc levels, BMI (Body Mass Index) and blood pressure (assessed at baseline and 12-weeks). All measures of blood glucose were measured by the Glucose Oxidase method (BaiAnyi, Bayer HealthCare LLC.).

### Statistical analysis

Our analyses were based on the intention-to-treat principle. Statistical analysis was performed using SPSS 19.0 Software (SPSS Inc., Chicago, IL, USA). Repeated measurements were applied to compare primary and secondary outcomes. First, we compared the taVNS and sham taVNS groups; then, we separately compared real and sham taVNS with the no-treatment control population, to further assess and isolate the treatment effects of taVNS and sham taVNS.

## Results

**Figure 3:**
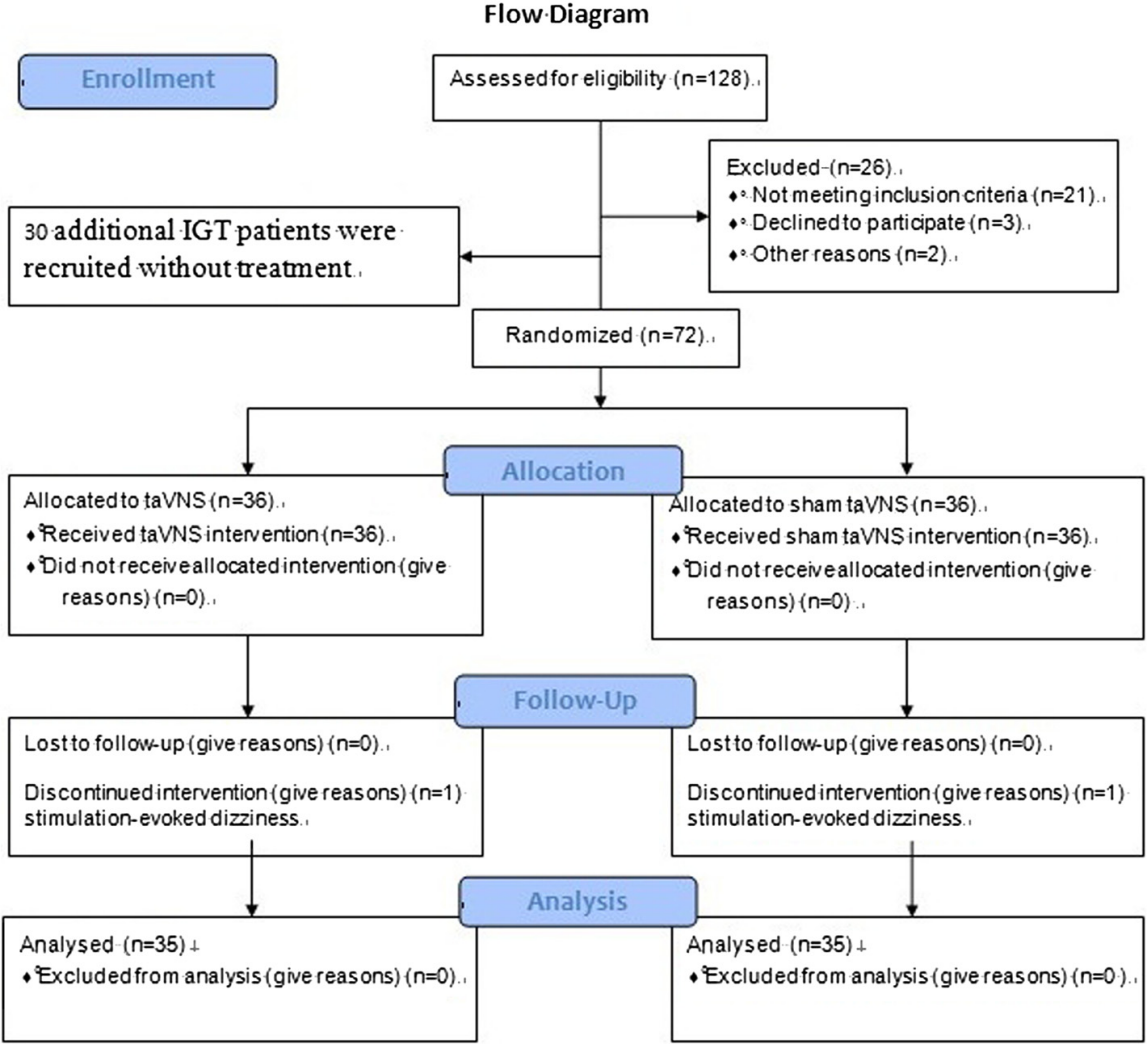
The Flow diagram shows detailed information regarding recruited and excluded participants.

A total of 102 participants completed the study (72 females, 54.4 ± 7.4 years (mean ± SD), range: 33–68). Two female patients (one in the taVNS group and one in the sham taVNS group) dropped out of the study due to stimulation-evoked dizziness. The symptoms were relieved after stopping treatment. All other participants reported following all treatment instructions with a reported compliance rate of 98.04%. Figure 3 details the recruited and excluded participants.

### Comparison between the taVNS and sham taVNS

Comparison by Independent Samples t-test showed that the two groups did not differ in age (*t*(70) = 1.51, p = 0.14), weight (*t*(70) = −0.83, p = 0.41) systolic blood pressure (*t*(70) = 1.42, p = 0.16), diastolic blood pressure (*t*(70) = 0.22, p = 0.16), or BMI (t(70) = 64.07, p = 0.61) at baseline (Table 1). The gender distribution also did not differ significantly across groups (χ^2^ (2, n = 72) =3.29, p = 0.07). Measures of FPG (*t*(70) = 0.3, p = 0.77), 2hPG (*t*(70) = 1.96, p = 0.054) and HbAlc (*t*(70) = 1.12, p = 0.27) similarly did not differ between groups at baseline.

**Table 1:** Baseline characteristics across three groups

**Group**	**Age**	**Gender (M/F)**	**Height**	**Weight**	**BMI**
**taVNS**	55.3 (7.1)	7/29	1.6 (.06)	63.9 (11.7)	24.5 (3.5)
**Sham taVNS**	52.3 (8.7)	14/22	1.7 (.08)	66 (10.1)	23.9 (2.6)
**Control**	55.7 (5.6)	9/21	1.6 (.07)	67.2 (9)	25.7 (3.4)

All values are presented as mean (SD).

**Figure 4:**
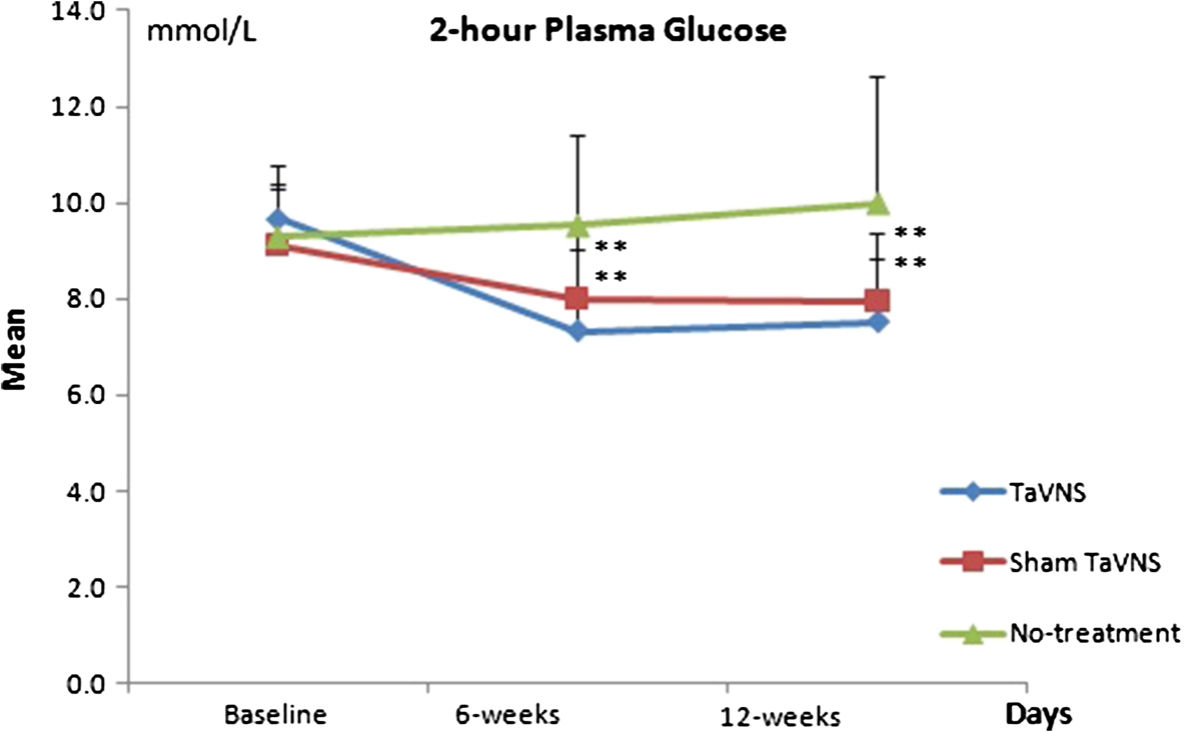
2-hour plasma glucose level changes among taVNS, sham taVNS and no-treatment control groups.

Comparison of the taVNS and sham taVNS groups using repeated measures analysis of variance (ANOVA) indicated a significant difference in 2hPG between groups over the course of the experiment (*F*(2) = 5.79, p = 0.004) (Figure 4 and Table 2). The decrease in 2hPG was significantly greater in the taVNS group compared to that in the sham taVNS group (Table 3). After adjusting for age, gender, and BMI, the effect remained significant (Table 2). Measures of FPG (*F*_*GG*_ (1.84) = 2.48, p = 0.093) and HbAlc (*F*(1) = 0.23, p = 0.63) did not differ significantly between the taVNS and sham taVNS groups over time in both crude analysis and after adjusting for age, gender, and BMI (Table 2). For FPG, Mauchly’s Test of Sphericity indicated that assumptions of sphericity were violated, thus Greenhouse Geisser corrected degrees of freedom were used.

**Table 2:** Comparison of 2-hPG, FPG and HbAlc between taVNS and sham taVNS groups

		**P-value**	**Lower 95% CI**	**Upper 95% CI**
**2hPG**	Crude	.004	8.02	8.522
	Adjusted	.006	8.015	8.526
**FPG**	Crude	.093	5.946	6.203
	Adjusted	.11	5.948	6.201
**HbAlc**	Crude	.63	6.057	6.282
	Adjusted	.681	6.056	6.283

Adjusted values reflect age, gender, and BMI as covariates.

**Table 3:** 2-hPG, FPG and HbAlc before and after the intervention across different groups

	**Ta-VNS**	**Sham taVNS**	**No-treatment**
**2hPG Baseline**	9.7 (1.2)	9.1(1.2)	9.3 (1.1)
**2hPG 6-weeks**	7.3 (1.8)^**^	8.0 (1.6)^**^	9.5 (1.9)
**2hPG 12-weeks**	7.5 (1.3)^**^	8.0 (1.4)^**^	10.0 (2.7)
**FPG Baseline**	6.2 (0.6)	6.3 (0.5)	6.5 (0.3)
**FPG 6-weeks**	5.9 (0.8)^**^	6.2 (0.8)	6.6 (0.8)
**FPG 12-weeks**	5.7 (0.6)^**^	6.2 (0.8)	6.9 (1.2)
**HbAlc Baseline**	6.3 (0.5)	6.2 (0.6)	6.2 (0.4)
**HBAlc 12-weeks**	6.1 (0.4)^*^	6.0 (0.4)^*^	6.3 (0.6)
**BMI Baseline**	24.5 (3.5)	23.9 (2.6)	25.7 (3.4)
**BMI 12-weeks**	24.1 (3.3)	23.5 (2.6)	25.4 (3.0)

Compared to No-treatment group, *p < 0.05, **p < 0.01.

All values are presented as mean (SD).

Further analysis of other secondary outcomes indicated that the taVNS and sham taVNS groups differed significantly in systolic blood pressure over time (*F*(1) = 4.21, p = 0.044). In the taVNS group, systolic blood pressure dropped from 123.69 ± 14.14 (mean ± SD) to 118.64 ± 13.34, while in the sham taVNS group, systolic blood pressure remained at 119 ± 12. No significant differences were observed for changes in diastolic blood pressure (*F*(1) = 0.75, p = 0.39) or BMI (*F*(1) = 0.069, p = 0.79).

### Comparison between taVNS, sham taVNS and no-treatment control

In this study, we added a separate no-treatment control group recruited from a free community clinic physical exam program. This group was included to better understand the natural fluctuation of outcomes in patients with IGT and to isolate the pure treatment effects from other naturally occurring factors.

Analysis of variance indicated that the three experimental groups did not differ in age (*F*(2) = 1.95, p = 0.15), weight (*F*(2) = 0.85,p = 0.43), diastolic blood pressure (*F*(2) = 1.05, p = 0.37), gender distribution (χ^2^(2, n = 102) = 3.29, p = 0.2), or BMI (*F*(2) = 2.96, p = 0.057) at baseline. Measures of FPG (*F*(2) = 2.86, p = 0.06), 2hPG (*F*(2) = 2.03, p = 0.14) and HbAlc (*F*(2) = 1, p = 0.37) also did not differ between groups at baseline. There was, however, a significant difference in systolic blood pressure (*F*(1) = 1.02, p = 0.01).

Repeated measures ANOVA between the taVNS and no-treatment control indicated significant differences in FPG (*F*(2) = 10.62, p < 0.001), 2hPG (*F*(2) = 25.18, p < 0.001) and HbAlc (*F*(1) = 12.79, p = 0.001) between groups over the course of the 12 weeks. All effects remained significant after adjusting for age, gender, and BMI (Table 4).

**Table 4:** Comparison of 2-hPG, FPG and HbAlc between taVNS and no-treatment control groups

		**P-value**	**Lower 95% CI**	**Upper 95% CI**
**2hPG**	Crude	<.001	8.553	9.238
	Adjusted	<.001	8.546	9.241
**FPG**	Crude	<.001	6.164	6.473
	Adjusted	<.001	6.162	6.476
**HbAlc**	Crude	.001	6.123	6.346
	Adjusted	.002	6.124	6.342

Adjusted values reflect age, gender, and BMI as covariates.

Analysis of other secondary outcomes, with comparison between the taVNS and no-treatment control groups, indicated that there were no significant differences between the two groups in systolic blood pressure (*F*(1) = 0.99, p = 0.32). diastolic blood pressure (*F*(1) = 1.27, p = 0.27), or BMI (*F*(1) = 0.003, p = 0.96) over time.

Repeated measures ANOVA between the sham taVNS and no-treatment control groups showed that the two groups differed significantly in their levels of 2hPG (*F*_*GG*_(1.72) = 10.51, p < 0.001) and HbAlc (*F*(1) = 5.94, p = .018) over the course of the experiment. Measures of both 2hPG and HbAlc increased over the 12 weeks in the control group, and decreased over the course of the 12 weeks in the sham taVNS treatment group. After controlling for age, gender, and BMI, only the effect for change in 2hPG remained significant (Table 5).

**Table 5:** Comparison of 2-hPG, FPG and HbAlc between the sham taVNS and no-treatment control groups

		**P-value**	**Lower 95% CI**	**Upper 95% CI**
**2hPG**	Crude	<.001	8.63	9.336
	Adjusted	.003	8.636	9.294
**FPG**	Crude	.055	6.293	6.603
	Adjusted	.3	6.294	6.587
**HbAlc**	Crude	.018	6.051	6.285
	Adjusted	.07	6.052	6.247

Adjusted values reflect age, gender, and BMI as covariates.

Analysis of other secondary outcomes between the sham taVNS and no-treatment control indicated that there were no significant differences between the two groups in systolic blood pressure (*F*(1) = 1.44, p = 0.24), diastolic blood pressure (*F*(1) = 0.047, p = 0.83), or BMI (*F*(1) = 0.024, p = 0.88) over time.

### Safety

According to the patients’ booklet reports, we found that 2 patients reported feeling dizzy during or after applying treatment. All participants recovered fully from the adverse events after stopping the treatment.

## Discussion

As the epidemic of diabetes continues to rise, it becomes essential to develop and implement cost-effective preventative therapeutic strategies [8]. In this study, We compared the efficacy of taVNS and sham taVNS in patients with IGT. Results showed that compared with sham taVNS, taVNS significantly reduced the two-hour glucose tolerance. In addition, we found that taVNS significantly decreased systolic blood pressure over time (*F*(1) = 4.21, p = 0.044) as compared with controls who received no treatment. Taken together, our results suggest that taVNS demonstrates potential use as a preventive treatment for pre-diabetes or IGT.

The metabolic syndrome, which includes a constellation of risk factors such as obesity, elevated lipids, and elevated glucose and blood pressure, has increasingly drawn attention from investigators; this syndrome is regarded as both a disease and a risk factor for other major diseases such as cardiovascular disease and Alzheimer’s disease (AD) [9]. These diseases not only harm our well-being and longevity, but also have a tremendous economic impact [15]. Prevention and treatment of these disorders require a considerable number of medical resources. The discovery of a simple and cost-effective method of controlling these disorders may significantly benefit society.

Previous studies have suggested that the vagus nerve plays an important role in maintaining metabolic homeostasis. These studies further suggest that efferent vagus nerve-mediated cholinergic signaling controls immune function and proinflammatory responses via the inflammatory reflex. Deregulation of metabolism and immune function are associated with chronic inflammation, which acts as a critical step in the pathogenesis of insulin resistance and type 2 diabetes mellitus [9,16].

In a previous study, investigators [17] found that metabolic syndrome had an independent inverse association with the vagal component of high-frequency heart rate variability. In another study, Licht and colleagues [18] found that decreased parasympathetic and increased sympathetic activities are associated with increased likelihood of metabolic syndrome. Specifically, they found that lower respiratory sinus arrhythmia (RSA), a measure of parasympathetic activity in which high RSA reflects high parasympathetic activity, was associated with glucose levels and systolic blood pressure. Taken together, these studies suggest that low vagus nerve activity may underlie elevated glucose levels, which provides the biological mechanism of modulating IGT using taVNS.

Anatomical studies have shown that the ear is the only place on the surface of the human body where there is afferent vagus nerve distribution (12; 13). Thus, direct stimulation of the afferent nerve fibers on the ear can regulate the activity of the vagus nerve, which can further regulate metabolic homeostasis. In this study, we found that taVNS reduced both IGT and systolic blood pressure. This result suggests that taVNS has the potential for use as a therapeutic method for IGT and pre-diabetes, as well as potentially for other metabolic syndromes.

In this study, we found that both taVNS and sham taVNS significantly reduced the 2-hour glucose tolerance and levels of HbAlc, indicating that sham taVNS could also produce a significant, and desirable, modulatory effect on glucose levels. We speculate that this may be due to the strong electrical stimulation at the sham point extending to the adjacent area of the ear, where there is vagus nerve distribution. In addition, sham taVNS may produce these placebo effects by influencing patient lifestyle. Patients were asked to apply the postprandial treatment twice daily, which might have reminded them to pay more attention to food intake and levels of daily activity.

There are several limitations in this study. First, treatments in the study were self-administered by the patients, and thus patient compliance may have influenced the observed results. To enhance compliance, all patients were required to complete daily entries into a diary that was checked during assessments. More importantly, however, this self-administration method provides direct evidence for the feasibility of wide application of the method used within the study. This shows promise in significantly reducing the expenses associated with such a treatment. Secondly, the treatment was only 12 weeks in duration, thus the results obtained only represent its short or mid-term effects. Further study is warranted to evaluate the long-term effects of this treatment option. Thirdly, we only measured blood glucose level at three time points (the baseline, after 6 weeks, and after 12 weeks) for statistic analysis. Since blood glucose levels may be affected by the food intake of the previous day, a more frequent measurement in the future study may provide more reliable information for the influence of treatment on blood glucose level. Finally, the no-treatment control group was not included in the randomization scheme, as were the other two treatment groups. The participants constituting the control group were also recruited from a different population. However, the purpose of this group was to add another layer of control to explore the effects of the different treatments; thus, it should not influence the conclusions of this study.

## Conclusions

In summary, this pilot study demonstrates that taVNS can significantly reduce two-hour glucose tolerance and systolic blood pressure. As a simple, cost-effective therapeutic method with mild side effects, it demonstrates great potential in the treatment of pre-diabetes and associated metabolic disorders.

## Competing interests

The authors declare that they have no competing interests.

## Authors’ contributions

PJR designed the trail and was the principal clinical research investigator. PJR, FH, wrote the manuscript with JK. XL generated the random allocation sequence. FH, HM, CHW, XL, XZ, JXD were in charge of the recruitment and treatment of patients in the hospital and collected the data. FH prepared the figures. JK, SC and RBS were responsible for the data analysis and edited the manuscript with BZ. All authors read and approved the final manuscript.

## Pre-publication history

The pre-publication history for this paper can be accessed here:

http://www.biomedcentral.com/1472-6882/14/203/prepub
