# Erratum to: Effect of transcutaneous auricular vagus nerve stimulation on impaired glucose tolerance: a pilot randomized study

**DOI:** 10.1186/s12906-016-1190-1

**Published:** 2016-07-13

**Authors:** Feng Huang, Jianxun Dong, Jian Kong, Hongcai Wang, Hong Meng, Rosa B. Spaeth, Stephanie Camhi, Xing Liao, Xia Li, Xu Zhai, Shaoyuan Li, Bing Zhu, Peijing Rong

**Affiliations:** Institute of Acu-Mox, China Academy of Chinese Medical Sciences, 16# Nanxiao Street, Dongzhimennei, Beijing, 100700 China; Beijing University of Chinese Medicine, Beijing, 100029 China; Beijing Hospital of T.C.M Affiliated to Capital University of Medicine Sciences, Beijing, 100010 China; Department of Psychiatry, Massachusetts General Hospital, Harvard Medical School, Boston, MA USA; Department of Psychology, Endicott College, Beverly, MA USA; Institute of Basic Research in Clinical Medicine, China Academy of Chinese Medical Sciences, Beijing, 100700 China

## Erratum

In response to concerns raised by readers of this paper [1], the authors have accepted that corrections are required in order to clarify aspects which are unclear and/or potentially misleading as presently reported. The corrections do not affect the underlying results of the study, but may affect the interpretation of the findings.

**Abstract**

The Methods section in the Abstract should more explicitly state the nature of this study as not simply a randomized control trial, but a trial with a related observational study.

The original text reads as:

In this pilot randomized clinical trial, we compared the efficacy of transcutaneous auricular vagus nerve stimulation (taVNS) and sham taVNS on patients with IGT. 72 participants with IGT were single-blinded and were randomly allocated by computer-generated envelope to either taVNS or sham taVNS treatment groups. In addition, 30 IGT adults were recruited as no-treatment control so as to monitor the natural fluctuation of glucose tolerance in IGT patients.

The revised text reads as:

In this pilot randomized clinical trial with an additional observation group, we first compared the efficacy of transcutaneous auricular vagus nerve stimulation (taVNS) and sham taVNS on patients with IGT. 72 participants with IGT were single-blinded and were randomly allocated by computer-generated envelope to either taVNS or sham taVNS treatment groups. Then, an additional 30 IGT adults were recruited separately as a control population and not assigned treatment so as to monitor the natural fluctuation of glucose tolerance in IGT patients.

**Methods**

The authors wish to clarify the statistical analyses and comparisons performed in their Methods sections.

The original text reads as:

Statistical analysis

Our analyses were based on the intention-to-treat principle. Statistical analysis was performed using SPSS 19.0 Software (SPSS Inc., Chicago, IL, USA). Repeated measurements were applied to compare primary and secondary outcomes. First, we compared the taVNS and sham taVNS groups; then, we separately compared real and sham taVNS with the no-treatment control population, to further assess and isolate the treatment effects of taVNS and sham taVNS.

The revised text reads as:

Our analyses were based on the intention-to-treat principle, and only subjects who had completed a data set were included in data analysis. Statistical analysis was performed using SPSS 19.0 Software (SPSS Inc., Chicago, IL, USA). Repeated measurements were applied to compare primary and secondary outcomes. First, we compared the taVNS and sham taVNS groups; then, we separately compared real and sham taVNS with the no-treatment control condition (data from observational group) to further assess and isolate the treatment effects of taVNS and sham taVNS. For repeated measurements, Mauchly’s Test of Sphericity were applied, if assumptions of sphericity were violated, Greenhouse Geisser corrected degrees of freedom were applied. In addition, we also repeated the above analysis to include age, gender and BMI as covariates to adjust for the effects of factors.

**Results**

The results section should more explicitly separate the comparisons between groups from solely the randomised controlled trial, and the observational comparisons which made use of both the randomised and observational data.

The original text reads as:

Comparison between the taVNS and sham taVNS

Comparison by Independent Samples t-test showed that the two groups did not differ in age (t(70) = 1.51, p = 0.14), weight (t(70) = −0.83, p = 0.41) systolic blood pressure (t(70) = 1.42, p = 0.16), diastolic blood pressure (t(70) = 0.22, p = 0.16), or BMI (t(70) = 64.07, p = 0.61) at baseline (Table 1). The gender distribution also did not differ significantly across groups (χ2 (2, n = 72) =3.29, p = 0.07). Measures of FPG (t(70) = 0.3, p = 0.77), 2hPG (t(70) = 1.96, p = 0.054) and HbAlc (t(70) = 1.12, p = 0.27) similarly did not differ between groups at baseline. Comparison of the taVNS and sham taVNS groups using repeated measures analysis of variance (ANOVA) indicated a significant difference in 2hPG between groups over the course of the experiment (F(2) = 5.79, p = 0.004) (Figure 4 and Table 2). The decrease in 2hPG was significantly greater in the taVNS group compared to that in the sham taVNS group (Table 3). After adjusting for age, gender, and BMI, the effect remained significant (Table 2). Measures of FPG (FGG (1.84) = 2.48, p = 0.093) and HbAlc (F(1) = 0.23, p = 0.63) did not differ significantly between the taVNS and sham taVNS groups over time in both crude analysis and after adjusting for age, gender, and BMI (Table 2). For FPG, Mauchly’s Test of Sphericity indicated that assumptions of sphericity were violated, thus Greenhouse Geisser corrected degrees of freedom were used. Further analysis of other secondary outcomes indicated that the taVNS and sham taVNS groups differed significantly in systolic blood pressure over time (F(1) = 4.21, p = 0.044). In the taVNS group, systolic blood pressure dropped from 123.69 ± 14.14 (mean ± SD) to 118.64 ± 13.34, while in the sham taVNS group, systolic blood pressure remained at 119 ± 12. No significant differences were observed for changes in diastolic blood pressure (F(1) = 0.75, p = 0.39) or BMI (F(1) = 0.069, p = 0.79).

**Table 2:** Comparison of 2-hPG, FPG and HbAlc between taVNS and sham taVNS groups. Adjusted values reflect age, gender, and BMI as covariates

Measurements		*P*-value
2hPG	Crude	.004
	Adjusted	.006
FPG	Crude	.093
	Adjusted	.11
HbAlc	Crude	.63
	Adjusted	.681

Comparison between taVNS, sham taVNS and notreatment control

In this study, we added a separate no-treatment control group recruited from a free community clinic physical exam program. This group was included to better understand the natural fluctuation of outcomes in patients with IGT and to isolate the pure treatment effects from other naturally occurring factors. Analysis of variance indicated that the three experimental groups did not differ in age (F(2) = 1.95, p = 0.15), weight (F(2) = 0.85,p = 0.43), diastolic blood pressure (F(2) = 1.05, p = 0.37), gender distribution (χ2(2, n = 102) = 3.29, p = 0.2), or BMI (F(2) = 2.96, p = 0.057) at baseline. Measures of FPG (F(2) = 2.86, p = 0.06), 2hPG (F(2) = 2.03, p = 0.14) and HbAlc (F(2) = 1, p = 0.37) also did not differ between groups at baseline. There was, however, a significant difference in systolic blood pressure (F(1) = 1.02, p = 0.01). Repeated measures ANOVA between the taVNS and no-treatment control indicated significant differences in FPG (F(2) = 10.62, p < 0.001), 2hPG (F(2) = 25.18, p < 0.001) and HbAlc (F(1) = 12.79, p = 0.001) between groups over the course of the 12 weeks. All effects remained significant after adjusting for age, gender, and BMI (Table 4). Analysis of other secondary outcomes, with comparison between the taVNS and no-treatment control groups, indicated that there were no significant differences between the two groups in systolic blood pressure (F(1) = 0.99, p = 0.32). diastolic blood pressure (F(1) = 1.27, p = 0.27), or BMI (F(1) = 0.003, p = 0.96) over time. Repeated measures ANOVA between the sham taVNS and no-treatment control groups showed that the two groups differed significantly in their levels of 2hPG (FGG(1.72) = 10.51, p < 0.001) and HbAlc (F(1) = 5.94, p = .018) over the course of the experiment. Measures of both 2hPG and HbAlc increased over the 12 weeks in the control group, and decreased over the course of the 12 weeks in the sham taVNS treatment group. After controlling for age, gender, and BMI, only the effect for change in 2hPG remained significant (Table 5). Analysis of other secondary outcomes between the sham taVNS and no-treatment control indicated that there were no significant differences between the two groups in systolic blood pressure (F(1) = 1.44, p = 0.24), diastolic blood pressure (F(1) = 0.047, p = 0.83), or BMI (F(1) = 0.024, p = 0.88) over time.

**Table 4:** Comparison of 2-hPG, FPG and HbAlc between taVNS and no-treatment control groups. Adjusted values reflect age, gender, and BMI as covariates

		*P*-value
2hPG	Crude	<.001
	Adjusted	<.001
FPG	Crude	<.001
	Adjusted	<.001
HbAlc	Crude	.001
	Adjusted	.002

**Table 5:** Comparison of 2-hPG, FPG and HbAlc between the sham taVNS and no-treatment control groups. Adjusted values reflect age, gender, and BMI as covariates

	Measurements	*P*-value
2hPG	Crude	<.001
	Adjusted	.003
FPG	Crude	.055
	Adjusted	.3
HbAlc	Crude	.018
	Adjusted	.07

The revised text reads as:

Comparison between the taVNS and sham taVNS (results based on data from randomized trials)

Comparison by Independent Samples t-test showed that the two groups did not differ in age (t(70) = 1.51, p = 0.14), weight (t(70) = −0.83, p = 0.41) systolic blood pressure (t(70) = 1.42, p = 0.16), diastolic blood pressure (t(70) = 0.22, p = 0.16), or BMI (t(70) = 64.07, p = 0.61) at baseline. The gender distribution also did not differ significantly across groups (X2 (2, n = 72) =3.29, p = 0.07). Measures of FPG (t(70) = 0.3, p = 0.77), 2hPG (t(70) = 1.96, p = 0.054) and HbAlc (t(70) = 1.12, p = 0.27) similarly did not differ between groups at baseline.

Comparison of the taVNS and sham taVNS groups using repeated measures analysis of variance (ANOVA) indicated a significant difference in 2hPG between groups over the course of the experiment (F(2) = 5.79, p = 0.004) (Figure 3 and Table 2). The decrease in 2hPG was significantly greater in the taVNS group compared to that in the sham taVNS group (Table 3). After adjusting for age, gender, and BMI, the effect remained significant (Table 2). Measures of FPG (FGG (1.84) = 2.48, p = 0.093) and HbAlc (F(1) = 0.23, p = 0.63) did not differ significantly between the taVNS and sham taVNS groups over time in both crude analysis and after adjusting for age, gender, and BMI (Table 2). For FPG, Mauchly’s Test of Sphericity indicated that assumptions of sphericity were violated, thus Greenhouse Geisser corrected degrees of freedom were used.

Further analysis of other secondary outcomes indicated that the taVNS and sham taVNS groups differed significantly in systolic blood pressure over time (F(1) = 4.21, p = 0.044). In the taVNS group, systolic blood pressure dropped from 123.69 ± 14.14 (mean ± SD) to 118.64 ± 13.34, while in the sham taVNS group, systolic blood pressure remained at 119 ± 12. No significant differences were observed for changes in diastolic blood pressure (F(1) = 0.75, p = 0.39) or BMI (F(1) = 0.069, p = 0.79).

Comparison between taVNS, sham taVNS and no-treatment control (results based on randomized trial and additional observational group)

In this study, we added a separate no-treatment control group recruited from a free physical exam program at a community clinic. This group was included to better understand the natural fluctuation of outcomes in patients with IGT and to isolate the pure treatment effects from other naturally occurring factors.

Analysis of variance indicated that the three experimental groups did not differ in age (F(2) = 1.95, p = 0.15), weight (F(2) = 0.85,p = 0.43), diastolic blood pressure (F(2) = 1.05, p = 0.37), gender distribution (χ2(2, n = 102) = 3.29, p = 0.2), or BMI (F(2) = 2.96, p = 0.057) at baseline. Measures of FPG (F(2) = 2.86, p = 0.06), 2hPG (F(2) = 2.03, p = 0.14) and HbAlc (F(2) = 1, p = 0.37) also did not differ between groups at baseline. There was, however, a significant difference in systolic blood pressure (F(1) = 1.02, p = 0.01).

Repeated measures ANOVA between the taVNS and no-treatment control indicated significant differences in FPG (F(2) = 10.62, p < 0.001), 2hPG (F(2) = 25.18, p < 0.001) and HbAlc (F(1) = 12.79, p = 0.001) between groups over the course of the 12 weeks. All effects remained significant after adjusting for age, gender, and BMI (Table 4).

Analysis of other secondary outcomes, with comparison between the taVNS and no-treatment control groups, indicated that there were no significant differences between the two groups in systolic blood pressure (F(1) = 0.99, p = 0.32), diastolic blood pressure (F(1) = 1.27, p = 0.27), or BMI (F(1) = 0.003, p = 0.96) over time.

Repeated measures ANOVA between the sham taVNS and no-treatment control group showed that the two groups differed significantly in their levels of 2hPG (FGG(1.72) = 10.51, p < 0.001) and HbAlc (F(1) = 5.94, p = .018) over the course of the experiment. Measures of both 2hPG and HbAlc increased over the 12 weeks in the control group, and decreased over the course of the 12 weeks in the sham taVNS treatment group. After controlling for age, gender, and BMI, only the effect for change in 2hPG remained significant (Table 5).

Analysis of other secondary outcomes between the sham taVNS and no-treatment control group indicated that there were no significant differences between the two groups in systolic blood pressure (F(1) = 1.44, p = 0.24), diastolic blood pressure (F(1) = 0.047, p = 0.83), or BMI (F(1) = 0.024, p = 0.88) over time.

Tables 2, 4 and 5

A repeated measures analysis of variance (ANOVA) at three time points was applied, and the p values in Tables 2, 4, and 5 corresponding to the test of significance of the group by time interaction. Since these do not test the significance at a signal time point, the authors accept that confidence intervals are not appropriate for these hypotheses. The confidence intervals have therefore been removed from Tables 2, 4 and 5:

Corrected tables:

Discussion

The authors accept that the nature of their study as a pilot was unclear. The section of their Discussion dealing with limitations has therefore been amended.

The original text reads as:

Secondly, the treatment was only 12 weeks in duration, thus the results obtained only represent its short or mid-term effects. Further study is warranted to evaluate the long-term effects of this treatment option.

The revised text reads as:

Secondly, as a pilot study, the treatment was only 12 weeks in duration. Thus the results obtained only represent its short or mid-term effects. Further study is warranted to evaluate the long-term effects of this treatment option.
